# Effects of neuromuscular versus stretching training performed during the warm-up on measures of physical fitness and mental well-being in highly-trained pubertal male soccer players

**DOI:** 10.1371/journal.pone.0318318

**Published:** 2025-02-21

**Authors:** Achraf Hammami, Walid Selmi, Abdelkader Mahmoudi, Yassine Negra, Anis Chaouachi, David G. Behm, Urs Granacher, Raouf Hammami

**Affiliations:** 1 Higher Institute of Sport and Physical Education of Ksar-Said, Manouba University, Tunis, Tunisia; 2 Tunisian Research Laboratory ‘Sports Performance Optimization’ (CNMSS-LR09SEP01), National Center of Medicine and Science in Sports (CNMSS), Tunis, Tunisia; 3 Research Laboratory (LR23JS01) “Sport Performance, Health & Society”, Tunis, Tunisia; 4 School of Human Kinetics and Recreation, Memorial University of Newfoundland, St. John’s, Newfoundland and Labrador, Canada,; 5 Department of Sport and Sport Science, Exercise and Human Movement Science, University of Freiburg, Freiburg, Germany; University of Study of Bari Aldo Moro, ITALY

## Abstract

While there is ample evidence on the effects of neuromuscular training (NMT) and stretching training on selected measures of physical fitness in young athletes, less is known on the mental well-being effects. Here, we aimed to examine the effects of NMT versus stretching training (ST) performed during the warm-up and active control (CG) on selected physical fitness measures and mental well-being in highly-trained male pubertal soccer players. A secondary aim was to investigate associations between training-induced changes in physical fitness and mental well-being. Forty-six pubertal participants aged 12.2 ± 0.6 years were randomly allocated to NMT, ST, or CG. The eight-weeks NMT included balance, strength, plyometric, and change-of-direction (CoD) exercises. ST comprised four weeks of lower limbs static stretching followed by four weeks of dynamic stretching. The CG performed a soccer-specific warm-up. Training volumes were similar between groups. Pre-, and post-training, tests were scheduled to assess dynamic balance (Y-balance test), 15-m CoD speed, power (five-jump-test [FJT]), cognitive (CA), somatic anxiety (SA), and self-confidence (SC). Findings showed significant group-by-time interactions for all physical fitness measures (d =  1.00-3.23; p < 0.05) and mental well-being (d = 0.97-1.08; p < 0.05) tests. There were significant pre-post changes for all tested variables (d = 0.69-4.23; p < 0.05) in favor of NMT but not ST and CG. Pooled data indicated significant moderate correlations between training-induced performance changes in FJT and SA (r = −0.378, p < 0.05), FJT and SC (r = 0.360, p < 0.05) and 15-m CoD and SA (r = 0.393, p < 0.01). NMT but not ST or CG resulted in improved measures of physical fitness and mental well-being in highly-trained pubertal male soccer players. NMT performed during the warm-up is a safe and effective training method as it exerts positive effects on physical fitness and self-confidence as well as the coping of anxiety in highly-trained male pubertal soccer players.

## Introduction

Youth soccer players’ physical fitness (e.g., balance, muscle strength, power, change-of-direction speed [CoD]) [1] and mental well-being (i.e., self-esteem, life-happiness, satisfaction) [2] play crucial roles in accommodating the general and/or specific demands of training and match play. Indeed, there is evidence that measures of physical fitness (CoD speed, muscle strength, power) [3] and mental well-being (stress and anxiety, self-confidence) [4] have the potential to differentiate between soccer players of different expertise levels. Understanding the specific links between cognitive anxiety, somatic anxiety, and self-confidence is crucial in youth soccer. Cognitive anxiety is associated with negative thoughts, and somatic anxiety involves physical symptoms such as enhanced muscle activation which can negatively influence physical fitness and decision-making [5]. Conversely, self-confidence has been shown to mitigate the effects of anxiety, enhancing focus and resilience [6]. By examining these connections, coaches can develop intervention programs to manage anxiety and build confidence, promoting physical fitness and mental well-being in young soccer players. Accordingly, physical fitness and mental well-being should be developed from an early age to enable youth soccer players to perform their sport physically and mentally healthy [7].

For physical fitness development, Granacher et al. [8] designed a conceptual strength and conditioning model to assign adequate neuromuscular training (NMT) methods according to the individual’s resistance training skill competency and maturity status. The inherent advantage of NMT is that it is a multimodal training regime including balance, linear sprint and CoD speed, muscle strength and power exercises to improve physical fitness [9,10] and reduce injury occurrence [2]. In a meta-analysis including nine studies, Williams et al. [10] examined the effects of NMT on measures of physical fitness in youth aged 8-18 years and found positive NMT effects for muscle strength, power, speed, and balance. Using moderator analyses, the authors observed larger NMT effects for younger (< 13.8 years) compared with older youth (> 13.8 years) and for boys compared with girls.

Moreover, Granacher et al. [8] summarized the available literature on NMT effects in youth and deduced dose-response relations. The authors postulated that a training frequency of at least two sessions per week, a training duration of at least eight weeks, and a progressive intensity of 3-8 on an OMNI scale appear adequate to improve performance in youth. In addition, Fernandez-Fernandez et al. [11] performed an intervention study and contrasted the effects of NMT applied as a warm-up program versus a traditional dynamic warm-up on measures of physical fitness and sport-specific performance in young elite tennis players. Following eight weeks of training, the authors reported larger performance improvements for NMT compared with the traditional warm-up for linear sprint speed, vertical jump height, medicine ball throw distance, and serve velocity. While there is ample evidence on the fitness effects of NMT in the general youth population [9] and in youth soccer players [3,12], less is known on the effects of NMT on mental well-being. This is particularly important for youth soccer athletes who attend youth soccer academies from an early age, experiencing selection pressure during training and competition. In this regards, McLafferty et al. [13] investigated the effects of a 24-week NMT program on mental well-being in youth athletes and observed significant improvements in overall mood and marked reduction in anger, confusion, and mental tension. Similarly, Marinkovic et al. [14] postulated that NMT has the potential to enhance mental well-being in youth athletes aged 13 years.

Stretching training (ST) is a traditional and widely applied conditioning program implemented in the warm-up of soccer training [15,16] and it has proven to enhance measures of physical fitness and mental well-being [8,10]. Improvements in physical fitness such as increased muscle strength, power and refined motor control, can have an impact on psychological states such as confidence and anxiety in youth soccer players [5]. Research indicates that enhanced physical fitness can positively influence self-confidence and reduce anxiety by fostering a sense of competence and control in young athletes [17]. Understanding these relationships is essential for developing training programs that promote physical and psychological development, finally leading to athletic success and mental well-being in young soccer players. For instance, Kilit et al. [18] examined the effects of different stretching methods (static vs dynamic vs combined static and dynamic ST) on measures of speed and agility in young tennis players aged 13.4 ±  0.3 years. The authors showed that dynamic and combined static and dynamic ST induced significant 10-m and 20-m linear sprint speed and agility improvements compared with static ST. In another study, Sharififar et al. [19] investigated the effects of ST on anxiety and attitude in youth. The authors reported significant training-related improvements in attitude and mental health. Hence, there is preliminary evidence that different types of exercise (NMT, ST) improve both, physical fitness and mental well-being in youth and youth athletes.

Furthermore, the implementation of NMT and ST during the warm-up phase is essential for youth soccer players to enhance physical fitness and reduce the risk of sustaining injuries. There is ample evidence showing that NMT has the potential to improve balance, muscle strength and power, and change-of-direction speed [8]. At the same time, NMT has proven to reduce injury occurrence [20]. There is evidence showing that ST enhances flexibility and prepares athletes to perform dynamic activities with sufficient range of motion, ultimately reducing the likelihood of sustaining muscular injuries [21]. Thus, incorporating these training modalities into the warm-up of soccer training ensures that young players are physically prepared and better protected during training and competition.

Given that NMT [10,22] and ST [15,16] can easily be implemented in the warm-up program of soccer-specific training and that particularly ST has been widely used in the past, the primary aim of this study was to examine the effects of NMT versus ST and an active control (CG) on selected components of physical fitness (i.e., dynamic balance, muscle power, and CoD speed) and mental well-being (i.e., somatic and cognitive anxiety, self-confidence) in highly-trained male pubertal soccer players. A secondary aim was to investigate potential associations between training-induced changes in physical fitness and mental well-being. We hypothesized that particularly NMT and to a lesser extent static and dynamic ST included in the warm-up of soccer-specific training sessions has the potential to improve selected measures of physical fitness [9,10] and mental well-being [13,14] in highly-trained male pubertal soccer players.

## Materials and methods

### Participants

The sample size estimation was computed using G*Power software (version 3.1.6). Based on findings from a related study [23] examining the effects of repeated sprint training on somatic and cognitive anxiety, self-confidence, rating of perceived exertion (RPE) and repeated sprint ability in elite young soccer players, an a priori power analysis with a type I error of 0.01 and 90% statistical power was computed. The analysis indicated that 45 players would be needed to observe significant interaction effects for cognitive anxiety (Cohen’s f = 0.34). Accordingly, a total of 46 highly trained male pubertal soccer players from the same soccer team were recruited to participate in this study on March 26^th^ 2024. Athletes were randomly assigned to NMT (n = 16), ST (n = 15), or active CG (n = 15) (Table 1). All participants practiced systematic soccer training for at least 4-5 years prior to study participation and competed in the top-level national soccer division. Of note, all groups followed the same soccer training program under the supervision of the same coaches. NMT and ST included two weekly training sessions implemented in the warm-up of regular soccer-training sessions (Table 2). Participants’ biological maturity status was estimated based on the maturity offset method using the prediction equation of Moore et al. [24]. Before participation in this study, the subjects were given a letter that included written information about the study and a request for consent from the parents to allow their children to participate in the study. Legal representatives and subjects provided informed consent after thorough explanation of the objectives and scope of this project, the procedures, risks, and benefits of the study. The study was conducted according to the latest version of the Declaration of Helsinki and the protocol was fully approved by the Local Clinical Research Ethics Committee (Personal Protection Committee) under the following code (N°: 0225//2024) before the commencement of any assessments. None of the participating players had a history of psychological and musculoskeletal, neurological, or orthopedic disorders six months prior to the start of the study.

**Table 1:** Anthropometrics of the examined study cohort according to group allocation.

	NMT (n = 16)	ST (n = 15)	CG (n = 15)
Age (years)	12.4 ± 0.6	12.1 ± 0.3	12.2 ± 0.7
Height (cm)	158.3 ± 8.7	149.3 ± 7.1	152.5 ± 7.5
Body fat % (cm)	12.7 ± 3.4	11.5 ± 4.7	13.3 ± 2.8
Body mass (kg)	40.6 ± 7.7	37.4 ± 5.7	37.9 ± 4.6
Maturity offset (years)	−0.9 ± 0.5	−1.5 ± 0.3	−1.3 ± 0.6
Predicted APHV (years)	13.3 ± 0.5	13.6 ± 0.4	13.5 ± 0.3

Notes: Data are presented as means and standard deviations; NMT: neuromuscular training; ST: stretching training; CG: control group; APHV: Age at peak-height-velocity. Athletes were randomly assigned to NMT (n = 15), ST (n = 15), or an active CG (n = 15).

**Table 2:** Exemplified NMT, ST and CG warm-up training programs.

Type of training	NMT	ST	CG
**Warm-up**	15 min (NMT) 3 × 5 to 10 repetitions of (2 sessions/week) 8 weeks of balance, strength/power, sprint and CoD exercises	15 min (ST); (2 sessions/week) 4 weeks of static stretching followed by 4 weeks of dynamic stretching	15 min soccer training; (2 sessions/week) 8 weeks of soccer-specific ball passing skills
**Soccer specific training**	Technical and tactical drills (35 min) Small-sided games with or without goal (35 min)		
**Cool down**	5 min Jogging and stretching	5 min Jogging and stretching	5 min Jogging and stretching
**Total training volume**	90 min	90 min	90 min

Notes: Technical drills: ball control, ball pass and dribbling, feints and moves, positional rotations, combination passing, crossing, ball kicking etc. Tactical drills: Soccer-specific defending drills for specific defending positions such as centre defenders, outside backs etc.

Soccer-specific attacking drills for specific attacking positions such as centre forwards, wingers, attacking mid-fielders etc. NMT: neuromuscular training; ST: stretching training; CG: control.

### Procedures

One week prior to the start of the study, a familiarization session was scheduled to allow players to become acquainted with the applied tests and exercises. Participants of NMT and ST received instructions on the proper exercise techniques. CG performed standardized and already familiarized exercises. The same test sequence was applied during pre and post-tests. More specifically, athletes performed the dynamic balance test followed by the five-jump test (FJT), the CoD-speed test and the cognitive, somatic anxiety tests and the self-confidence test. This test sequence was selected to perform balance tests in unfatigued state and the physically more demanding tests (FJT, CoD-speed) thereafter. Before testing, all participants conducted a standardized 10-minute warm-up which consisted of submaximal running (e.g., skipping, hip in or out), balance exercises (e.g., forward or backward beam walking, single-leg stance on unstable devices) and landing drills (e.g., snap downs, 1-leg drop squat). All tests were separated by a 5–10 minutes rest period. The rest between test trials was three minutes. The best out of two trials was used for further statistical analyses, except for the cognitive, somatic anxiety and the self-confidence tests. For these tests, only one test trial was performed.

### Anthropometrics and body composition

Athletes’ body height and mass were collected using a wall-mounted stadiometer (Florham Park, NJ) and an electronic scale (Baty International, West Sussex, England), respectively. The sum of skinfolds was assessed using the Harpenden’s skinfold calipers. Body measurements were conducted according to Deurenberg et al. [25] who reported similar prediction errors between adults and adolescents. Thereafter, biological maturity was evaluated non-invasively using chronological age and body height for male youth athletes as input parameters for a regression equation to subsequently predict the maturity offset. (Maturity offset =  27.999994 +  [0.0036124 ×  age ×  height]) [24].

### Physical fitness tests

#### Dynamic balance.

Dynamic balance was assessed using the Y-balance test (YBT) according to a previously described protocol [22], which has been shown to be reliable for youth soccer players (ICC =  0.92) [26]. Testing was conducted barefoot. Participants stood on the dominant leg with the most distal aspect of their big toe on the center of the footplate from the YBT kit. The participants were then asked to push the reach-indicator block with the free limb in the anterior, posterior medial, and posterior lateral directions while maintaining their single-limb stance on the central footplate [27]. Participants were not allowed to lift the heel of the stance leg during the test performance. Maximal reach distances were recorded to the nearest 0.5 cm using the YBT kit. The trial was repeated if participants moved the stance leg or failed to return the reaching foot to the starting position. A composite score (CS-YBT) was calculated and considered as the dependent variable using the following formula: CS-YBT (%) =  ([maximum anterior reach distance +  maximum posteromedial reach distance +  maximum posterolateral reach distance]/[leg length x 3}) ×  100.

#### Muscle power.

As a proxy of muscle power, the FJT was applied in accordance with recommendations provided by Sabato et al. [28] From an upright standing position, with both feet flat on the ground, players tried to cover as much horizontal distance as possible with five forward bouncing strides by alternating left-leg and right-leg ground contacts. The covered distance was measured to the nearest 1 cm using a tape measure. Previously, test-retest reliability was described as high with an ICC =  0.91 for youth soccer players [29].

#### Change-of-direction (CoD) speed.

**Fig 1:**
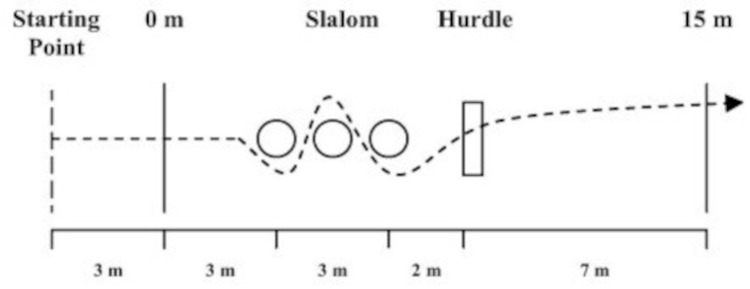
Schematic representation of the 15-m change-of-direction speed test. **Notes:** Players were instructed to perform 3-m linear accelerations before the initial set of photocell gates where they entered a 3-m slalom section marked by three aligned pylons (16-cm of height), placed 15 m apart and then cleared a 0.5-m hurdle placed beyond the third pylon.

CoD speed was tested using the 15-m CoD speed test. Players were instructed to perform 3-m straight accelerations before the initial set of gates where they entered a 3-m slalom section marked by three aligned pylons (16-cm of height), placed 15 m apart and then cleared a 0.5-m hurdle placed beyond the third pylon (Fig 1) [30]. An excellent test–retest reliability has been reported for the 15 m-CoD speed test with an ICC value of 0.93 [30].

### Mental well-being tests

#### Tests for the assessment of anxiety and self-confidence.

Before the intervention started, all participants used the adapted Arabic translation of the competitive state anxiety inventory-2 CSAI-2, using a total of 13 items as previously validated by Boudhiba et al. [31]. The CSAI-2 is a widely used tool designed to assess the multidimensional nature of state anxiety in athletes, with a particular focus on competitive settings. It evaluates three key components: i) cognitive anxiety, ii) somatic anxiety, and iii) self-confidence. Cognitive anxiety reflects athletes’ worries and negative thoughts about performance, somatic anxiety pertains to the physiological symptoms of anxiety, such as increased heart rate or enhanced muscle activation, and self-confidence represents the athlete’s belief in their ability to perform successfully.

The CSAI-2 provides valuable insights into how these factors interact and influence players’ performance and physical fitness, offering a robust framework for tailoring interventions aimed at reducing anxiety and enhancing self-confidence in young athletes [32]. For instance, participants are asked to indicate “how do you feel right now” for each item on a 4-point Likert scale ranging from “not at all” to “very much so.” Examples of the cognitive anxiety items include “I am concerned about this competition”, and “I am concerned about choking under pressure”. These items differ from the somatic anxiety statements such as “I feel nervous” or “I feel tense in my stomach”. The self-confidence subscale includes items such as “I feel at ease”, and “I am confident I can meet the challenge”. Each of the three subscales has 13 items, which are summed to get a score representing the level of intensity the athlete is feeling for each component of anxiety, and for self-confidence about performing. The reliability and validity of the translated CSAI-2 using 13 items were reported by Boudhiba et al. [31] with α = 0.85.

### Exercise programs

Both NMT and ST programs were conducted during the 2024 pre-season period, from August to October, with both groups participating in two weekly sessions. While NMT focused on improving selected components of physical fitness such as muscle strength and power, ST aimed at improving flexibility and range of motion of different lower limb joints to prepare athletes for subsequent soccer-specific exercises. Both programs promoted players’ overall athletic performance by targeting physical fitness and mental well-being.

#### Neuromuscular training (NMT).

The NMT was undertaken for eight weeks with two weekly sessions, each lasting 15 minutes. NMT was implemented in the warm-up of a soccer-specific training session and included five exercises to improve balance, strength/power, linear sprint and CoD speed with 3 sets and 5 to 10 repetitions each and a rest of 60–120 seconds between sets and exercises [12]. The RPE was adjusted every two weeks using a 0-10 OMNI scale. At the start of the intervention, a RPE of three on the OMNI scale was targeted. During weeks 3-4, the RPE was increased to five on the OMNI scale. During weeks 5 to 6, the RPE was increased to 7. In weeks 7-8, a RPE of nine was targeted. NMT exercises were conducted on the soccer pitch. A more detailed description of the program is displayed in Table 3.

**Table 3:** Design of the eight weeks neuromuscular training program (NMT).

	Weeks 1–2	Weeks 3–4	Weeks 5–6	Weeks 7–8
**Balance**	Single leg stance balance 3 sets of 5 repetitions	Single leg stance balance then controlling the ball around the body with the other leg. 3 sets of 5 repetitions	Single leg balance stance: 3 sets of 5 repetitions	Acceleration of 2-meters and vertical jumping before standing on single leg stance passing the ball with the dominant leg, then non-dominant leg. 3 sets of 5 repetitions
**Strength**	Body mass half squat and body mass single leg squat 2 sets of 10 reps Nordic hamstring exercises 1 set of 5 repetitions	Half squat and side lunges with a 2 kg medicine ball 3 sets of 8 repetitions Nordic hamstring exercises 2 sets of 5 reps	Bulgarian split squats and 4 direction lunges with a 2 kg medicine ball 3 sets of 10 reps Nordic hamstring exercises 2 sets of 8 repetitions	Bulgarian squats and 4 direction lunges with a 2 kg medicine ball 3 sets of 10 reps Nordic hamstring exercises 2 sets of 8 repetitions
**Power (plyometrics)**	Standing long jump 2 sets of 10 repetitions	Side to side ankle hops over the hurdle 3 sets of 8 repetitions	Double and one-legged lateral jumps over the hurdle. 3 sets of 10 repetitions	Cone hops with 180° side-cuts. 3 sets of 10 repetitions
**Change-of-direction speed**	Pre-planned lateral shuttle run of 3-meters (right and left or left and right) back and forth, then pass the ball.	Stop the ball (4 balls) after dribbling and following multiple changes of direction in a 4-m square. The total distance was 18 m.	Touch cones placed in a hexagon (2 m side length) with pre-planned order (N°1, N°2, N°3 and N°4) and then pass the ball. The total distance was 20 m.	Two meter acceleration with ball then slalom with the ball between 3 cones and finally pass the ball, and after that performing a 5-m acceleration without the ball. The total distance was 10-m.

Notes: Neuromuscular training comprised balance, strength, agility, and plyometric exercises. At the end of each training week, coaches tested whether athletes were able to perform two more repetitions per set. Once this was achieved, the intensity or difficulty/complexity of exercise was enhanced during NMT. NMT: neuromuscular training.

#### Stretching training (ST).

The ST protocol included four weeks of static stretching followed by four weeks of dynamic stretching and was performed for 15 minutes during the warm-up of a soccer-specific training session. The following muscles were exercised: m. gastrocnemius, m. gluteals, hamstrings, quadriceps, and adductors, abductors. During the first four weeks, participants performed a series of static stretching exercises where the stretching was carried out with three sets and a 40 second stretch duration per muscle, ten seconds rest between sets and with no rest between muscle groups. The intensity of the stretching exercises was the point of subjective discomfort. After the first four training weeks, participants executed a dynamic stretching program with three sets and 10, 20, 30, and 40 seconds stretch duration during weeks 5, 6, 7 and 8, respectively (Table 4). Movement velocity was slow during dynamic stretching in order to avoid muscular reflex activities due to fast stretches. ST intensity was defined using the 100% of the point of discomfort (POD) of the stretched muscle(s) [33,34].

**Table 4:** Design of the eight weeks static and dynamic stretching training program.

	Muscles groups	Week 1	Week 2	Week 3	Week 4
**SS**	Gastrocnemius Quadriceps Hamstring Adductors Abductors Gluteals	3 x 40 (s) with 10 s rest per muscle, 10 s rest between sets	3 x 40 (s) with 10 s rest per muscle, 10 s rest between sets	3 x 40 (s) with 10 s rest per muscle, 10 s rest between sets	3 x 40 (s) with 10 s rest per muscle, 10 s rest between sets
**DS**		**Week 5**	**Week 6**	**Week 7**	**Week 8**
		3 x 10 (s), 10 s rest between sets	3 x 20 (s), 10 s rest between sets	3 x 30 (s), 10 s rest between sets	3 x 40 (s), 10 s rest between sets

Notes: Stretching training included four weeks of static stretching followed by four weeks of dynamic stretching of the same muscles. For static and dynamic stretching, athletes were asked to slowly approach the stretch position at the point of discomfort. Static stretching involved holding a stretch in one position for 40 seconds. This had to be repeated three times per muscle group. For dynamic stretching, participants approached the stretch position at the point of discomfort and immediately released again.

#### Active control group (CG).

The CG participated in a 15-minute warm-up consisting of passing drills including short passes with a two-touch triangle and square passing patterns, as well as low-driven passes. These drills were designed to enhance the players’ technical proficiency and passing accuracy with players’ heart rates (HR) ranging between 120 to 140 beats per minute, ensuring an effective cardiovascular warm-up. After the warm-up, all three experimental groups followed the same soccer-specific training sessions, ensuring that the subsequent activities were identical, thereby controlling for training content, intensity, and volume. As a result, the total volume and intensity of training exposure (i.e., the overall time and intensity spent on training) was consistent across the groups, minimizing any potential differences in training load. The intervention was supervised by qualified coaches and experienced sport scientists, ensuring that the sessions were conducted safely and effectively, with professional oversight throughout the entire study period.

### Statistical analyses

Data are presented as group mean values and standard deviations (SD). After data normality was confirmed using the Shapiro-Wilk test, a MANOVA was applied to detect baseline between-group differences. A 3 x 2 ANOVA with repeated measures was computed on the factors groups (NMT, ST, CG) and time (pre, post) to determine training effects. Post-hoc tests with Bonferroni adjustments were conducted to identify group-specific pre- to post changes. Effect sizes for main time and group effects as well as group-by-time interactions were taken from the ANOVA output (partial eta squared transferred to Cohen’s d). Within-group Cohen’s d effect sizes (ES) were also calculated using the equation: d =  (mean post− mean pre−)/mean SD. d can be classified as small (0.00 <  d <  0.49), moderate (0.50 ≤  d <  0.80), and large (d >  0.80) [35]. Finally, Pearson’s correlation coefficients were computed to assess potential associations between selected measures of physical fitness and mental well-being in pubertal soccer players. Correlation coefficients were considered trivial (r <  0.1), small (0.1 <  r <  0.3), moderate (0.3 <  r <  0.5), large (0.5 <  r <  0.7), very large (0.7 <  r <  0.9), nearly perfect (0.9 <  r <  1.0), and perfect (r =  1.0) [36]. The level of significance was established at p <  0.05 and SPSS 20.0 was used for statistical analyses (SPSS Inc., Chicago, IL, USA).

## Results

All participants received treatments as allocated. The adherence rates were 92, 91, and 90% for NMT, ST, and CG participants, respectively. No significant between-group baseline differences were observed for any tested variable (Tables 1 and 5). Main effects of group, time, and group-by-time interactions are displayed in Table 5. No training or test-related injuries were recorded over the course of the study.

**Table 5:** Group-specific means and standard deviations for all outcome measures before (pre) and after (post) the intervention periods.

	Pre	Post	ANOVA outcomes			
			Delta change (%)	Group p-value (d)	Time p-value (d)	Group x time p-value (d)
CS-YBT (%)						
NMT (n = 16)	86.5 ± 13.5	99.1 ± 6.3	17.3	F = 2.25, p = 0.11 (0.64)	F = 19.02, p < 0.001 (1.33)	F = 5.30, p < 0.01 (1.00)
ST (n = 15)	85.3 ± 9.2	89.8 ± 8.5	5.4			
CG (n = 15)	87.4 ± 8.4	89.1 ± 5.4	2.3			
FJT (m)						
NMT (n = 16)	8.5 ± 0.9	10.1 ± 0.7	19.8	F = 12.61, P < 0.001 (1.53)	F = 101.23, p < 0.001 (1.70)	F = 55.98, p < 0.001 (3.23)
ST (n = 15)	8.1 ± 0.6	8.5 ± 0.3	5.3			
CG (n = 15)	8.6 ± 0.5	8.5 ± 0.5	0.6			
15-m-CoD test (s)						
NMT (n = 16)	3.7 ± 0.3	3.2 ± 0.2	13.3	F = 0.80, P = 0.454 (0.38)	F = 49.34, p < 0.001 (2.14)	F = 32.30, p < 0.001 (2.45)
ST (n = 15)	3.6 ± 0.5	3.6 ± 0.5	0.3			
CG (n = 15)	3.6 ± 0.3	3.5 ± 0.3	1.5			
Cognitive anxiety						
NMT (n = 16)	14.2 ± 3.7	11.3 ± 2.6	14.9	F = 0.57, P = 0.566 (0.32)	F = 18.84, p < 0.001 (1.32)	F = 5.11, p < 0.01 (0.97)
ST (n = 15)	15.7 ± 5.5	10.7 ± 2.2	24.6			
CG (n = 15)	14 ± 4.2	13.9 ± 3.7	0.9			
Somatic anxiety						
NMT (n = 16)	14.3 ± 3.7	10.2 ± 2.4	27.3	F = 0.64, P = 0.527 (0.35)	F = 12.60, p < 0.001 (1.08)	F = 5.93, p < 0.001 (1.04)
ST (n = 15)	13.9 ± 3.2	12.3 ± 4	6.5			
CG (n = 15)	13.3 ± 4.3	13.5 ± 2.6	8.5			
Self-confidence						
NMT (n = 16)	24.5 ± 5.6	33.8 ± 1.8	45.8	F = 8.69, p < 0.01 (1.27)	F = 31.81, p < 0.001 (1.71)	F = 6.34, p < 0.01 (1.08)
ST (n = 15)	23.7 ± 7.9	26.2 ± 2.9	23.1			
CG (n = 15)	22.5 ± 4.1	25.5 ± 3.6	15.3			

**Notes:** NMT: neuromuscular training; ST: stretching training; CG: Control group; CS-YBT: composite score during Y-balance test; FJT: five-time jump test; CoD: change of direction; d: Cohen’s d.

### Dynamic balance

**Fig 2:**
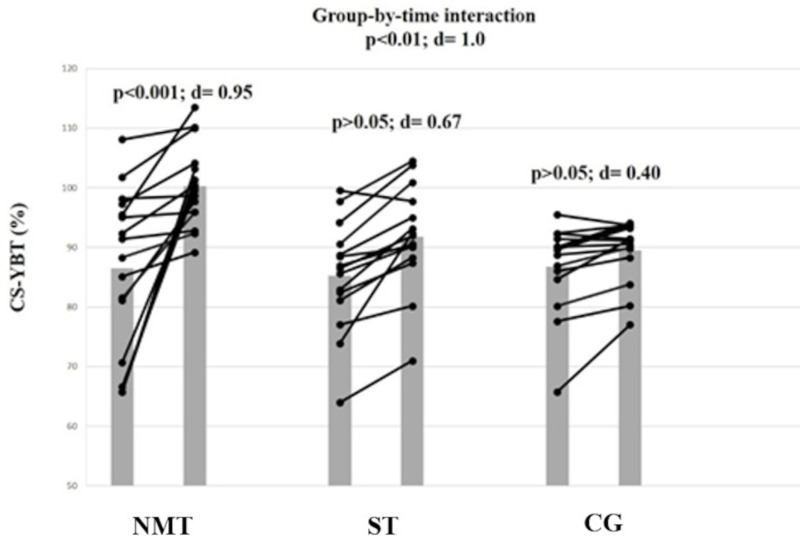
The figure displays intra-individual and group mean data illustrating the effects of three different training types implemented in the warm-up on dynamic balance in highly trained pubertal soccer players. NMT: neuromuscular training; ST: stretching training. CG: control group; CS-YBT: composite score during the Y-balance test.

A large magnitude group-by-time interaction effect was found for dynamic balance (F = 5.30, ES = 1.00, p < 0.01) (Table 4). Post-hoc analyses revealed that NMT but not ST or CG resulted in large improvements in dynamic balance (ES = 0.95; Δ17.3%, p < 0.01). For ST, the post-hoc analyses showed a moderate improvement for dynamic balance (ES = 0.67; Δ5.4%; p < 0.05; Fig 2).

### Muscle power

A very large magnitude group-by-time interaction effect was found for the FJT (F = 55.98, ES = 3.23; p < 0.001) (Table 5). Post-hoc analyses revealed that NMT but not CG resulted in a large FJT performance improvement (ES = 3.63; Δ19.8%, p < 0.01). For ST, the post-hoc analyses also showed a large magnitude FJT improvement (ES = 0.85; Δ5.3%, p < 0.01).

### Change-of-direction speed

A large group-by-time interaction effect was found for the 15-m CoD test (F = 32.30, ES = 2.45; p < 0.001) (Table 5). Post-hoc analyses revealed that NMT but not ST, or CG resulted in large magnitude 15-m CoD improvements (ES = 4.23; Δ13.3%, p < 0.001).

### Cognitive anxiety

The statistical analysis showed a large group-by-time interaction for cognitive anxiety (F = 5.11, ES = 0.97; p < 0.01) (Table 5). Post-hoc analyses showed that NMT but not ST or CG resulted in a moderate improvement in the cognitive anxiety score (ES = 0.69; Δ14.9%, p < 0.001).

### Somatic anxiety

We found a large group-by-time interaction for somatic anxiety (F = 5.93, ES = 1.04; p < 0.001) (Table 5). Post-hoc analyses revealed that NMT but not ST or CG resulted in a large somatic anxiety score improvement (ES = 1.78; Δ27.3%, p < 0.001).

### Self-confidence

**Fig 3:**
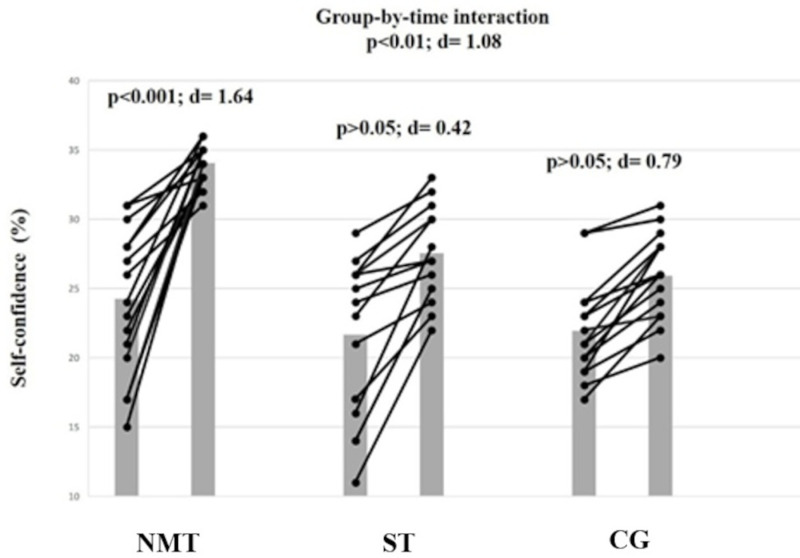
The figure displays intra-individual and group mean data illustrating the effects of three different training types implemented in the warm-up on self-confidence in highly-trained pubertal soccer players. NMT: neuromuscular training; ST: stretching training. CG: Control group.

The analysis revealed a large group-by-time interaction for self-confidence (F = 6.34, ES = 1.08; p < 0.01). The post-hoc analysis indicated that NMT but not CG or ST resulted in a large self-confidence score improvement (ES = 1.64; Δ45.8%, p < 0.001; Fig 3).

### Associations between training-induced performance changes in physical fitness and mental well-being

Findings from the correlational analyses from the whole study sample (N = 46) are displayed in Table 6. We contextualized the non-significant group-specific correlations in order to facilitate the interpretation of the associations between physical fitness performances and mental well-being variables. In this context, when pooling the data from all three groups, a statistically significant moderate correlation was found between training-induced performance changes in FJT and the somatic anxiety score (r = −0.378, p < 0.05). A statistically, significant moderate correlation was observed between performance changes between FJT and the self-confidence score (r = 0.360, p < 0.05) as well as between performance changes in the 15-m CoD test and the somatic anxiety score (r = 0.393, p < 0.01). We additionally analyzed group-specific associations between performance changes (pre, post) in physical fitness and mental well-being but could not detect any significant outcomes.

**Table 6:** Correlational analyses of pooled data from all three experimental groups between training induced pre-post changes (deltas ∆) in selected measures of physical fitness and psychological variables in youth soccer players (whole sample, N = 46).

		Δ Cognitive anxiety (%)	Δ Somatic anxiety (%)	Δ Self-confidence (%)
**Δ CS-YBT (%)**	**Pearson correlation**	0.014	−0.152	0.216
	**Sig. (2-tailed)**	0.924	0.313	0.150
	**N**	46	46	46
**Δ FJT (%)**	**Pearson correlation**	−0.008	−0.378**	0.360**
	**Sig. (2-tailed)**	,956	**0.010**	**0.014**
	**N**	46	46	46
**Δ CoD (%)**	**Pearson correlation**	0.045	0.393**	**−0.254**
	**Sig. (2-tailed)**	0.769	**0.07**	**0.089**
	**N**	46	46	46

**Notes:** CS-YBT: composite score during Y-balance test; FJT: five-time jump test; CoD: change of direction; d = Cohen’s d.

## Discussion

This study examined the effects of an eight week NMT versus ST implemented in the warm-up of soccer-specific training sessions and active control on selected measures of physical fitness and mental well-being in highly trained male pubertal soccer players. The main findings indicate that twice weekly NMT but not ST improved measures of dynamic balance, muscle power, CoD speed and somatic as well as cognitive anxiety and self-confidence in highly trained pubertal male soccer players. Moreover, when pooling the data from all three groups, significant moderate correlations were found between performance changes in FJT and SA (r = −0.378, p < 0.05). Significant moderate correlations were observed between performance changes in FJT and SC (r = 0.360, p < 0.05) as well as between 15-m CoD and SA (r = 0.393, p < 0.01). In addition, the correlational analyses did not reveal any significant group- specific associations between performance changes (pre-, post-test) in selected measures of physical fitness and mental well-being.

NMT is a multimodal exercise regime including balance, muscle strength, power, as well as linear sprint and CoD speed exercises. Our results indicated that NMT but not ST or CG resulted in large performance improvements in dynamic balance. This finding is in line with previous studies [3,12]. In accordance with the principle of training specificity [37], dynamic balance improved following NMT but not ST. This is most likely due to the fact that most NMT exercises include balance demands (e.g., squats, plyometrics and CoD). In addition, balance was specifically exercised (e.g., static and dynamic single leg stance) during NMT but not ST. Of note, strength exercises such as squats and plyometrics may induce balance demands due to the highly dynamic nature of these exercises. Previously, Hammami et al. [12] studied the effects of eight weeks of NMT versus active control on component of physical fitness in male youth soccer players. These authors reported large NMT-related balance improvements (i.e., CS-YBT). Accordingly, the dynamic nature of NMT exercises seems to provide an adequate and sufficient exercise stimulus to improve dynamic balance by promoting anticipatory postural adjustments [12].

This study further revealed improved muscle power (i.e., FJT) following NMT but not ST and CG. Again, the principle of training specificity may explain the observed finding because our NMT program involved exercises specifically aiming at enhancing muscle power [37]. Accordingly, the observed increases in horizontal jump performance (FJT) may have been primarily caused by the included plyometric exercises and the enhanced neural drive to the agonist muscles involved in horizontal jumping [12,38]. In fact, there is evidence that NMT has the potential to improve activities conducted in the stretch-shortening cycle in youth athletes [3].

Our results further showed improvements in 15-m CoD speed following NMT but not ST and CG. Several studies have investigated the effects of NMT on CoD speed in pubertal soccer players [3,39]. For instance, Zouhal et al. [39] examined the effects of a six week in-season NMT on CoD speed in youth male soccer players aged 17 years. These researchers reported greater improvements in CoD speed with 180° turns for the non-dominant and dominant legs in comparison with the active control group. The observed NMT effects on CoD speed may be related to increased neural drive to the prime movers (i.e., increased motor unit recruitment and firing frequency) and musculotendinous stiffness [40]. It should be acknowledged that the applied training duration of eight weeks might have been too short to improve musculotendinous stiffness which is why further research with more extended training durations is needed to examine musculotendinous adaptations following NMT in young athletes. A more likely reason for NMT-related improvements in CoD speed is a more efficient activation of the stretch-shortening cycle (i.e., rapid transition from eccentric to concentric muscle action) [38]. Accordingly, increased CoD speed performance following NMT, is speculated to be mainly the result of neural factors such as increased motor unit recruitment and firing frequency [41].

Of note, the reported training-related adaptations in selected measures of physical fitness appear to have translated to players’ psychological well-being. More specifically, we found less somatic anxiety and cognitive anxiety following NMT compared with ST. The latter can be explained by the fact that NMT may lead to a reduced emphasis on reactive cognitive control and concomitantly facilitating proactive cognitive control and processing efficiency [42]. Another reason could be related to a preference for reactive cognitive control strategies in youth soccer players [43]. Accordingly, the attentional control theory is an approach to anxiety and cognition representing a major development of Eysenck et al. [44] processing efficiency theory. In this context, anxiety impairs efficient functioning of the goal-directed attentional system and increases the extent to which processing is influenced by the stimulus-driven attentional system [45]. In addition to decreased attentional control, anxiety increases attention to threat-related stimuli. Adverse effects of anxiety on processing efficiency mainly depends on two central executive functions involving attentional control: inhibition and shifting [45]. However, anxiety may not impair performance effectiveness (quality of performance) when it leads to the use of compensatory strategies (e.g., enhanced effort; increased use of processing resources). Hence, proactive cognitive control depends on increased anxiety [46].

In this study, we observed that NMT resulted in improved self-confidence of pubertal male soccer players. This finding is in agreement with outcomes from a systematic review of Myer et al. [47] who observed that perceptions of health, physical competence and fitness can improve simply because there is a feeling that the body is adapting through exercise. In another study, Duncan et al. [48] showed that NMT enhanced self-esteem to a greater extent than regular physical education in boys aged 6 to 7 years. In addition, Sabato et al. [28] suggested strategies to enhance the physical and emotional health of young athletes. These authors showed that NMT is an effective tool to enhance self-esteem and prevent injuries in elite youth athletes. Findings from the current study indicate that highly-trained male young soccer players benefit from NMT in two ways. First, their physical fitness improves over a period of eight weeks. Second, their mental well-being increases as well, indicating that NMT should be implemented in the warm-up of soccer-specific training. The lack of psychological improvements with ST may be related to the lack of dynamic feedback related to soccer and their increased personal physical prowess. The positive feelings or impressions of increased strength (i.e., squats), speed (CoD and plyometrics), and power (plyometrics) have more direct attributes with soccer success possibly enhancing their personal self-image compared to the expected moderate less soccer specific improvements in range of motion.

Interestingly, when pooling the data from all three experimental groups, we observed significant associations between performance changes in physical fitness and mental well-being. In an earlier study, Reigal et al. [49] postulated that a regular engagement in physical exercise is important for a healthy upbringing in the form of improved physical fitness and mental well-being. Lang [50] described in his bio-informational theory that the perception of a training session or program, if meaningful to the participant, may evoke a different physiological and psychological response compared with a less representative session or program (i.e., ST).

While the correlational analyses revealed significant overall associations between pre to post-test changes in physical fitness and mental well-being, no significant group specific correlations were found, indicating that in general physical training is effective to improve mental well-being but not a specific type of training. This needs to be further investigated in future studies to verify our outcomes using larger study cohorts.

This study is not without limitations. First, we examined a sample of highly-trained male pubertal soccer players. Therefore, the results of this study are specific to the population under investigation. Second, it is important to consider the application of Bonferroni correction when multiple correlations are tested as realized in the present study. In case, Bonferroni correction has not been considered, the risk of reporting false positive findings increases, particularly when testing a large number of correlational analyses. Alternatively, the application of this adjustment enhances the statistical rigor and reduces the risk of overestimating the strength of the correlations between physical fitness and mental well-being variables. Furthermore, it is important to include and discuss additional statistical assumptions, such as homogeneity of variance and sphericity, which are essential for ensuring the robustness and accuracy of the statistical analyses. By addressing these assumptions in the results section, the interpretation of the statistical findings becomes more reliable and credible, reinforcing the accuracy of the conclusions drawn from the data. Third, to contextualize the non-significant group-specific correlations, it is important to consider the potential factors that may have influenced the lack of significant relationships between physical fitness and mental well-being outcomes according to groups. Non-significant results do not necessarily imply that there is no statistical correlation between the variables. Rather, they may suggest that the effect is too small to be detected given the usual sample sizes in (youth) elite sports, measurement methods, or variability within the groups. Additionally, it could indicate that other variables, not accounted for in the analysis, might play a more influential role in the observed outcomes. For example, individual differences in motor skill levels, psychological factors, or external conditions (such as training intensity or recovery time) could mediate or confound the magnitude of correlations being examined. By recognizing these possible influences, we can better interpret the non-significant findings and understand their context within the broader framework of the study. Further research with larger sample sizes or refined methods might help uncover more nuanced patterns, in order to facilitate the interpretation of the association between physical fitness and mental well-being variables. Finally, we evaluated the effects of NMT on physical fitness and mental well-being in comparison with a ST program and an active CG. However, we did not contrast the effects of NMT with other training types such as single-mode plyometric or strength training. Consequently, the outcomes of the present study are specific to the applied study design and should be interpreted with caution. Therefore, future studies could examine the effects of NMT in comparison to single-mode plyometric and/or strength training on measures of physical fitness such as muscle strength, power and mental well-being (e.g., feeling scale).

## Conclusions

This study demonstrated that eight weeks of NMT implemented in the warm-up of regular soccer training sessions has the potential to improve dynamic balance, horizontal jump, and CoD performances, self-confidence and somatic as well as cognitive anxiety to a larger extent than ST or a soccer-specific warm-up (CG) in highly-trained male pubertal soccer players. In addition, training-induced performance changes in selected measures of power and CoD appear to be associated with changes in somatic anxiety and self-confidence. Since we could not find group-specific associations, it seems that a general training effect accounts for the observed finding and not the effect of a specific training type. NMT is safe (no training related injuries) and effective to enhance physical fitness and reduce somatic as well as cognitive anxiety. However, further research is needed to compare the effects of NMT with single-mode plyometric and/or strength training in highly-trained male pubertal soccer players in order to verify and extend the findings of this study. Based on our findings, coaches as well as strength and conditioning professionals are advised to implement NMT as part of the warm-up in highly-trained male pubertal soccer players to enhance physical fitness and mental well-being.

## Abbreviations

1-RM: one repetition maximum
CA: cognitive anxiety
CG: control group
CoD: change of direction
CSAI-2: competitive state anxiety inventory-2
FJT: five jump test
NMT: neuromuscular training
RPE: rating of perceived exertion
SA: somatic anxiety
SC: self-confidence
ST: stretching training
YBT: Y-balance test
APHV: age at peak-height-velocity
SS: static stretching
DS: dynamic stretching
d: Cohen’s d.
